# Effect of the Molecular Weight of Carboxymethyl Cellulose on the Flotation of Chlorite

**DOI:** 10.3390/ma16093356

**Published:** 2023-04-25

**Authors:** Yanfei Chen, Yuanlin Chen, Lei Zhang

**Affiliations:** 1 School of Metallurgy and Environment, Central South University, Changsha 410083, China; yfeichen@126.com (Y.C.); yuanlinchen88@126.com (Y.C.); 2 School of Minerals Processing and Bioengineering, Central South University, Changsha 410083, China

**Keywords:** chlorite, carboxymethyl cellulose, entrainment, flocculation

## Abstract

The present study aimed to investigate the influence mechanism of carboxymethyl cellulose (CMC) on the flotation of fine chlorite. To this end, a series of flotation tests, sedimentation tests, and microscope analyses were conducted. Flotation tests revealed an inverse relationship between particle size and the recovery of chlorite, indicating that finer particles exhibited higher recovery rates. Moreover, it was observed that the recovery of fine chlorite was significantly associated with the water recovery (proportion of water entering the floated product to the weight of water in the initial flotation suspension) and a variety of frother types. Based on these findings, it can be inferred that froth entrainment may constitute a crucial component of the recovery mechanism underlying fine chlorite. Thus, reducing froth entrainment (the phenomenon of hydrophilic minerals entering floated products through foam water) is the key to depress chlorite flotation. Flotation tests indicate that fine chlorite recovered into froth products can be depressed effectively by CMC with a high molecular weight. The results of sedimentation tests and microscope analyses in the presence of CMC prove that CMC with a high molecular weight generates flocculation on fine chlorite particles while that with a low molecular weight does not. It is suggested that the depression of chlorite flotation may be attributed to the reduction in the entrainment resulting from the flocculation induced by CMC.

## 1. Introduction

As a prevalent magnesium silicate gangue mineral, chlorite is frequently associated with sulfides, such as copper-nickel sulfide [1,2]. Due to its low hardness, chlorite is susceptible to grinding, leading to the production of chlorite slimes which can negatively impact sulfide ore flotation via a phenomenon known as “slime coating” [3]. In addition, chlorite’s inherent floatability can result in it reporting to the concentrate during flotation, thereby reducing its grade and causing downstream processing issues, including heightened smelting costs [4,5].

Based on the above, the deleterious effect of magnesium silicate gangue minerals on the flotation of sulfide ores is mainly attributed to the “slime coating” of magnesium silicate gangue minerals on sulfide surfaces and the import of magnesium silicate gangue minerals into the concentrate. Numerous additives have been explored to mitigate the adverse impact of magnesium silicate gangue minerals. Sodium hexametaphosphate has been found to be effective in inhibiting magnesium silicate gangue minerals; however, its excessive use can generate phosphorus wastewater and result in environmental concerns [6]. In addition, oxalic acid has been utilized to alleviate the adverse effect of magnesium silicate gangue minerals on sulfide flotation [7]. Unfortunately, oxalic acid’s use is limited by its toxicity and potential health hazards upon ingestion. Thus, it is urgent to develop an ecological and efficient depressant for the flotation separation of sulfide from magnesium silicate gangue minerals.

It has been reported that the primary recovery mechanism of fine magnesium silicate gangue mineral particles (i.e., those with a diameter less than 20 μm), including chlorite, is primarily attributed to the entrainment facilitated by water recovery. In addition, the entrainment of minerals may be correlated with particle size [8]. However, most studies focus on the interaction between silicate and sulfide minerals rather than the entrainment, which also has a great influence on the concentrate grade of sulfide [9,10,11,12]. Thus, it is also very important to investigate the entrainment of chlorite in order to devise effective strategies for its elimination.

It is reported that the depression of silicate minerals from sulfide ore, especially when the entrainment of silicate minerals is serious, can be achieved with some polymers [13,14,15]. Carboxymethyl cellulose (CMC), the most commonly employed polysaccharide depressant, is known for its environmentally benign nature. Moreover, owing to its superior depressant performance, CMC finds extensive usage in the flotation separation of sulfide from silicate gangue minerals [16,17,18].

Many possible interaction mechanisms involving the possible contributions of electrostatic, chemical, hydrogen, and hydrophobic bonding between CMC and the surface of the magnesia-bearing mineral have been proposed [19,20]. Because the adsorbed amount of CMC depends on electrolyte concentration and pH, some studies have suggested that electrostatic interactions are involved in the adsorption process of CMC onto minerals [3,21,22]. Liu et al. believed that the nature of the interaction between mineral surfaces and natural polysaccharides, including CMC, is likely an acid/base interaction [23]. Fu et al. demonstrated that the adsorption of CMC on chlorite is significantly influenced by solution conditions [24]. The investigation conducted by Feng et al. revealed that the adsorption density of CMC onto chlorite was promoted by both copper ions and calcium ions. However, the underlying mechanisms of action for these two types of ions were found to differ [25]. In addition to silicate minerals, CMC also presents good depression performance for the flotation of other gangue minerals through selective adsorption [26,27,28].

CMC is known to be effective in reducing the floatability of minerals and its applications are diverse, with numerous systematic investigations having been conducted. However, the understanding of the various ways in which CMC influences chlorite with different particle sizes is currently inadequate, thereby impeding the broader application of CMC. Furthermore, it remains unclear whether the depressive effects of CMC are predominantly attributable to flocculation/dispersion [29]. Thus, the present study aims to explore the impact of flocculation/dispersion induced by CMC with varying molecular weights on the flotation of chlorite with the ultimate goal of facilitating the separation of sulfide from magnesium silicate gangue minerals during flotation.

## 2. Experimental Section

### 2.1. Samples and Reagents

**Figure 1:**
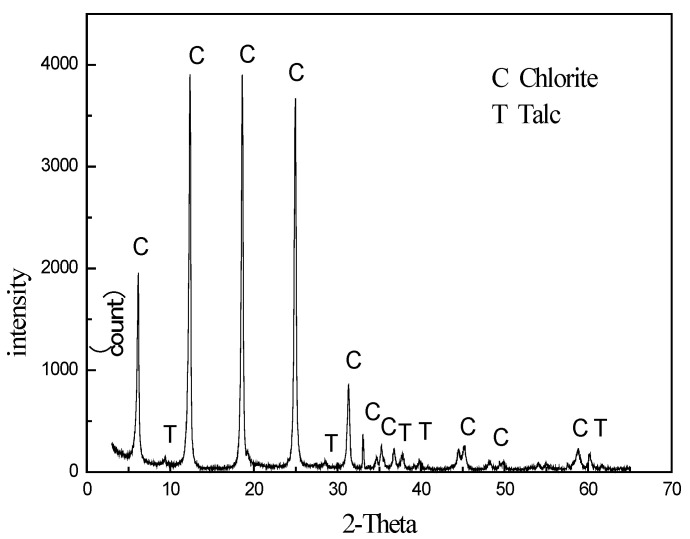
XRD of chlorite.

The chlorite utilized in the entire experiment was sourced from Haicheng, Liaoning Province, China. The XRD analysis (Figure 1) and chemical analysis (Table 1) data confirmed its high purity, with only trace amounts of talc present. The samples were subjected to dry grinding and screened to obtain three distinct size fractions: −100 + 75 μm, −75 + 38 μm, and −38 μm, which were collected separately for subsequent analyses.

CMC used for all tests was purchased from Aladdin Industrial Corporation, Shanghai, China. The molecular weight of the CMC we used in this work was 90,000, 250,000, 700,000, respectively. All three kinds of CMC were with the same degree of substitution, with 0.7. Terpilenol, MIBC (methyl isobutyl carbinol), and hexanol used as frothers, and were all obtained from Tianjin Guangfu Fine Chemical Research Institute, Tianjin, China. Hydrochloric acid (HCl) and sodium hydroxide (NaOH) were employed as pH modifiers and were procured from Tianjin Kermil Chemical Reagents Development Centre, Tianjin, China. All the chemicals were of analytical grade quality.

Stock solutions of CMC were prepared by dispersing a predetermined amount of solid into 100 mL of vigorously stirred cold distilled water and the stirring was continued for about 30 min until the CMC powders were dissolved completely. The solutions were freshly prepared each day. The HCl stock solution was prepared by adding a portion of known-weight HCl solution with a concentration of 36% to the appropriate amount of cold distilled water and stirring. NaOH stock solution was prepared by adding a portion of known-weight NaOH solid into the appropriate amount of cold distilled water and stirring until the NaOH powders were dissolved completely. Deionized double distilled water was used for all experiments.

### 2.2. Methods

#### 2.2.1. Flotation Tests

Flotation tests were performed using an XFG-type mechanical agitation flotation machine made by the Changchun Prospecting Machine Factory. In a typical single mineral flotation test, 2.0 g of mineral were added to 40.0 mL of distilled water, followed by conditioning. The pH of the mineral suspension was adjusted to the desired value using HCl or NaOH stock solution. Afterwards, CMC (if necessary) stock solution and a frother were added into the pulp and conditioned for 5 min and 1 min, respectively. Flotation was conducted for a duration of 4 min. Both the floated and sink products were collected, filtered, and subsequently dried before being weighed to facilitate the calculation of recovery. Each experiment was conducted in triplicate, and the average value was considered as the final result.

#### 2.2.2. Sedimentation Tests

Flocculation/dispersion of the chlorite was assessed via settling tests launched using a graduated cylinder. An amount of 0.1 g of chlorite powder was conditioned in a 100 mL beaker at the desired pH for 5 min, following which CMC stock solution was added and gentle stirring was carried out for an additional 5 min using a magnetic stirrer. The suspension was then transferred to a 100 mL cylinder and the water level was adjusted to 100 mL using distilled water. The cylinder was stoppered, inverted twenty times, and then allowed to remain in an upright position for a fixed duration of 10 min. The suspension in the upper 25 mL of the cylinder was siphoned out and measured using a WGZ-3(3A) type Scattering Turbidimeter fabricated by the Shanghai Xinrui Instrument Company. The degree of flocculation/dispersion of the suspension was assessed based on the turbidity of the supernatant liquor. Lower turbidity values were indicative of superior flocculation. Each test was repeated thrice, and the average value was considered as the final outcome.

#### 2.2.3. Microscope Analyses

Visual examination of the flocculation/dispersion state of fine chlorite was performed using a polarized optical microscope Leica DM4800. The procedure for the preparation of the slurry was identical to that mentioned in the sedimentation tests. A drop of slurry was dispensed onto a glass slide by pipette during the stirring of the slurry, following which the sample was examined using a microscope, which was fitted with a video camera.

## 3. Results and Discussion

### 3.1. The Flotation Behaviors of Chlorite with Different Particle Sizes

**Figure 2:**
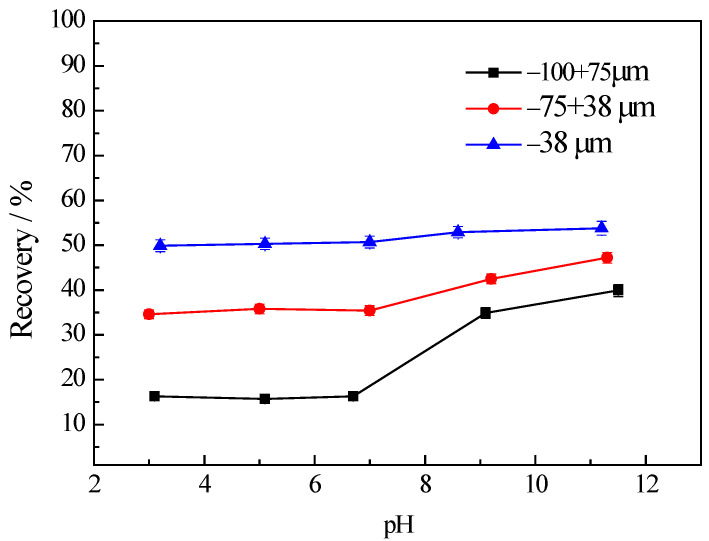
The recovery of chlorite in different size fractions with flotation condition of *C* (MIBC) = 8 mg/L.

Figure 2 illustrates the variation in the flotation recovery of chlorite with different particle sizes as a function of pH. It can be observed that the flotation recovery of the fine fraction (−38 μm) without using any collector is dramatically higher than the other two coarse fractions (−100 + 75 μm and −75 + 38 μm respectively) over the entire range of the pH values tested. Apparently, the recovery of chlorite increases as the particle size of chlorite decreases, which indicates the recovery mechanism of fine chlorite particles may be due to froth entrainment. Similar results were also obtained by Pietrobon et al. in their research [8]. Li et al. [30] believed that during the flotation process of fine-grained minerals hydrophilic minerals would be mechanically entrained into the concentrate, leading to a decrease in the concentrate grade, which has been a major issue in the flotation of fine-grained minerals. Kirjavainen et al. [31] studied the flotation of fine sericite and quartz in the absence of hydrophobic minerals and found that the entrainment of hydrophilic gangue minerals was influenced by the quality and shape of particles. The smaller particle sizes of minerals corresponded with the higher levels of entrainment. When the particle size of the minerals was close to the colloidal particle size, the entrainment ratio was mainly determined by the particle size and the entrainment ratio was close to 1.

### 3.2. The Recovery Mechanism of Fine Chlorite

**Figure 3:**
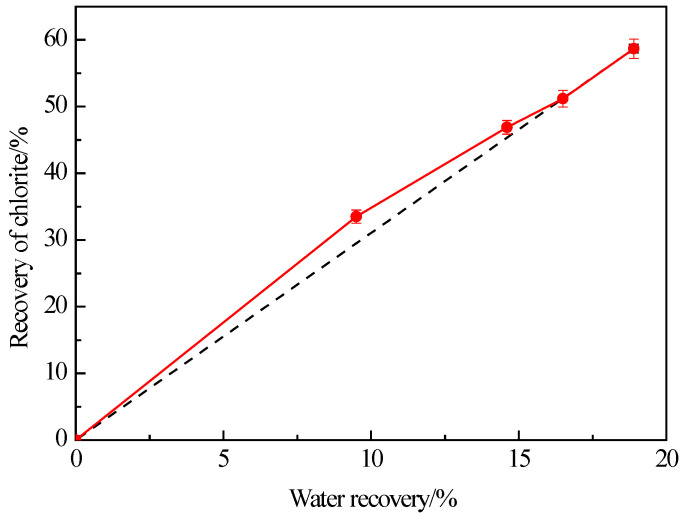
Effect of water recovery on the flotation recovery of −38 μm chlorite with flotation condition of pH = 9.0 ± 0.2, *C* (MIBC) = 8 mg/L.

In the flotation process, fine-grained minerals and hydrophilic minerals may be entrained into the concentrate by foam water. It is well known that the froth entrainment is closely linked to the water recovery (proportion of water entering the floated product to the weight of water in the initial flotation suspension) during flotation. Figure 3 illustrates the flotation recovery of −38 μm chlorite as a function of water recovery. It shows that at the beginning of flotation, the recovery of fine chlorite increases faster than the water recovery. The recovery of fine chlorite increases essentially linearly with water recovery at an acceptable error level. Many previous studies have confirmed a correlation between the entrainment recovery of gangue and the water recovery of floated products in flotation [32]. Li et al. [30] proposed that the recovery of hydrophilic gangue caused by froth entrainment is linear with the water recovery of concentrate in flotation, and the relationship between the two indices conformed to the following equation.

*Rg* = *e·Rw* (1)

where *Rg* is the recovery of hydrophilic gangue caused by froth entrainment, %; *e* is the entrainment factor of hydrophilic gangue; and *Rw* is the water recovery of the floated concentrate, %.

That is, the recovery of fine chlorite is directly proportional to the water recovery due to the froth entrainment of flotation.

**Figure 4:**
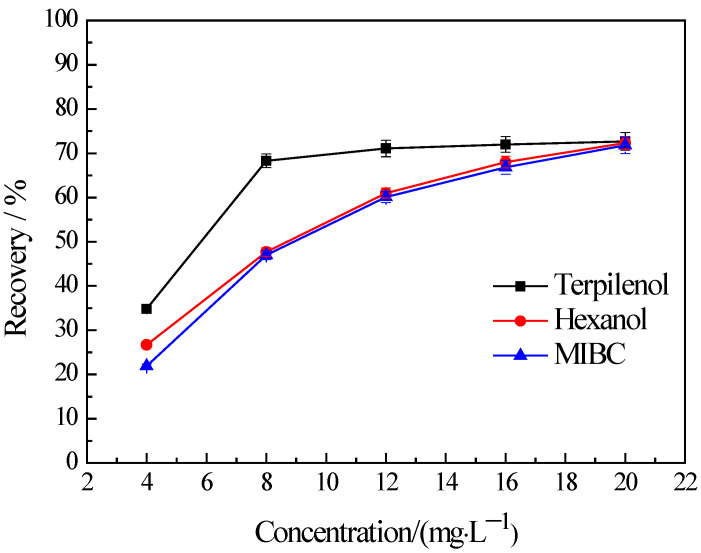
Effect of frother types on the flotation recovery of −38 μm chlorite with flotation condition of pH = 9.0 ± 0.2.

The water recovery in flotation is determined by the froth’s behaviors which are bound up with the properties of frothers and frothers almost do not change the hydrophobicity of mineral. Thus, in order to verify the froth entrainment, the flotation of −38 μm chlorite were carried out with three types of frothers. No collector was used in the flotation tests. The results are presented in Figure 4. It indicates that the flotation recovery of the fine chlorite rises in tandem with an increase in the concentration of a frother and this trend is observed across all three types of frothers used in the study, namely terpilenol, hexanol, and methyl isobutyl carbinol (MIBC). Figure 4 also shows that the recovery of flotation with terpilenol as a frother is obviously higher than that with hexanol and MIBC, and the recovery is almost the same when the last two were used as frothers.

From Figure 3 and Figure 4 we can conclude that the fine chlorite particles are recovered into froth products because of not only hydrophobicity but also froth entrainment. It is not surprising that the recovery of the fine chlorite is higher in the higher frother concentration since froth entrainment is closely related to foam volume, and the foam is rich with a high frother concentration. In addition, it is reasonable that there are significant differences among the recoveries of fine chlorite under different frothers. It has been reported that terpilenol presents better foam stability, higher foam viscosity, and higher water recovery than MIBC when they are used as frothers, but hexanol, as a kind of hexahydric alcohol, was similar to MIBC in foaming ability [33,34]. That is, the flotation recovery of the fine chlorite should be higher under terpilenol than MIBC for the higher water recovery, and the recovery under hexanol and MIBC should be the same in theory. The results presented in Figure 4 are confirmed by all evidence.

### 3.3. Influences of CMC on the Flotation of Chlorite with Different Particle Sizes

**Figure 5:**
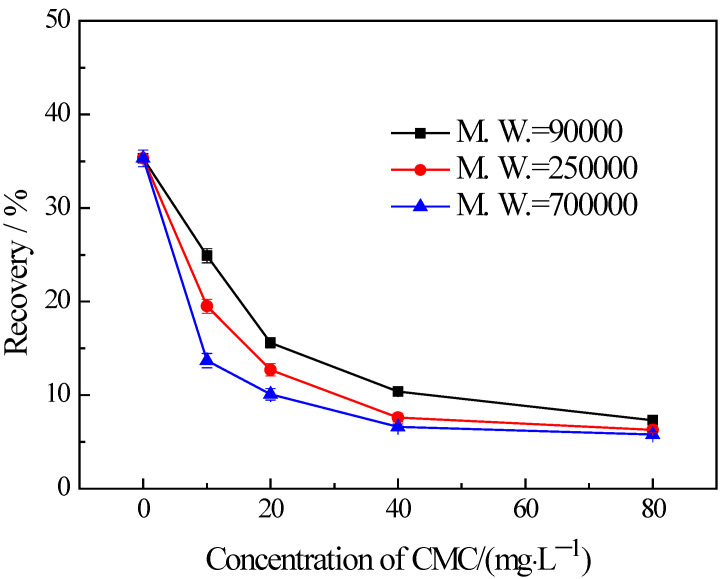
Flotation of −100 + 75 μm chlorite in the presence of different types of CMC with flotation condition of pH = 9.0 ± 0.2, *C* (MIBC) = 8 mg/L.

**Figure 6:**
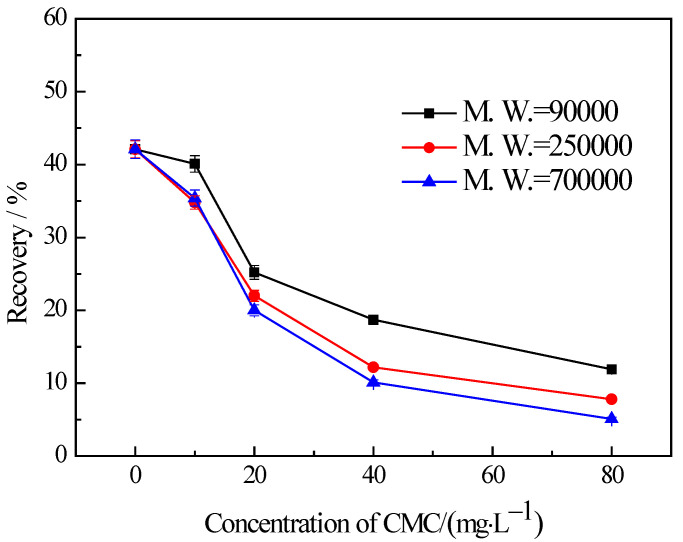
Flotation of −75 + 38 μm chlorite in the presence of different types of CMC with flotation condition of pH = 9.0 ± 0.2, *C* (MIBC) = 8 mg/L.

**Figure 7:**
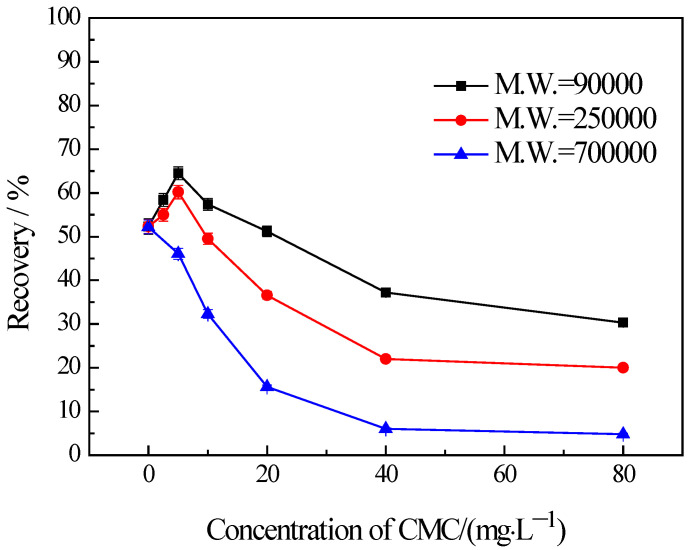
Flotation of −38 μm chlorite in the presence of different types of CMC with flotation condition of pH = 9.0 ± 0.2, *C* (MIBC) = 8 mg/L.

The findings from the flotation tests conducted on chlorite with different particle sizes in the presence of CMC are illustrated in Figure 5, Figure 6 and Figure 7. The results indicate the influences on the chlorite with different particle sizes caused by one certain type of CMC, especially the CMC with low molecular weight, is not the same. The chlorite in all the particle size fractions can be almost fully depressed by the CMC with a high molecular weight of 700,000. Additionally, the depressing effect on the −100 + 75 μm and −75 + 38 μm chlorite caused by the CMC with a molecular weight of 90,000 is as strong as that brought by the CMC with a molecular weight of 250,000 and 700,000. However, it is particularly noteworthy that, for the fine chlorite (−38 μm), the CMC with a low molecular weight of 90,000 is not an effective depressant, as is evidenced by the dramatically high recovery despite the highest dosage of this CMC. In fact, as shown in Figure 7, the CMC with a higher molecular weight gave a lower flotation recovery of the fine chlorite. Taking into account the flotation behaviors of chlorite observed in previous tests, it can be concluded that froth entrainment may contribute to the difficulties of the depression of fine chlorite by the low molecular weight CMC. In addition, it is interesting that in the flotation of the −38 μm chlorite (Figure 7) the two types of lower molecular weight CMC display a rise in recovery upon the addition of CMC at a low concentration.

### 3.4. Correlation between Froth Entrainment and Flocculation Caused by CMC

It has been mentioned above that the flotation recovery of the fine chlorite (−38 μm) in the presence of three types of CMC with the molecular weight of 90,000, 250,000, 700,000, respectively, are drastically different (Figure 7). In addition, the differences may be generated by froth entrainment in the flotation.

**Figure 8:**
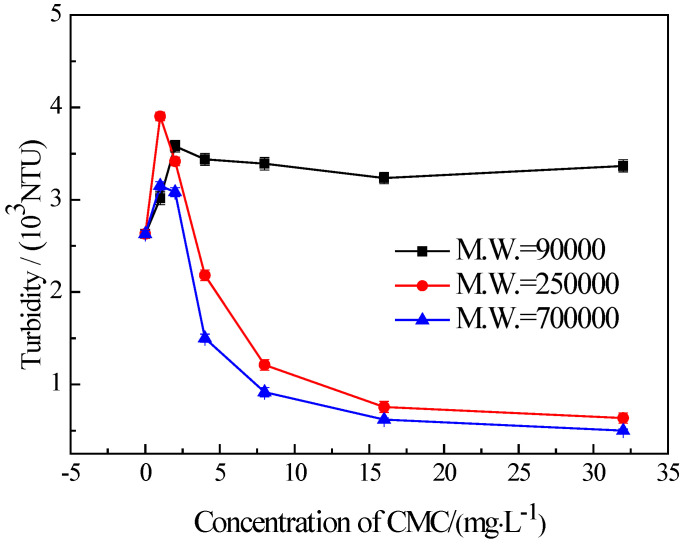
Settling behavior of −38 μm chlorite in the presence of different types of CMC with condition of pH = 9.0 ± 0.2.

It is well known that the recovery due to froth entrainment is also closely linked with fine particles. To investigate the influence of CMC molecular weights on the flotation of fine chlorite, a series of settling tests were carried out. The results are presented in Figure 8. It shows that the CMC with a molecular weight of 90,000 disperses the fine chlorite. On the other hand, the CMC with a higher molecular weight (250,000 and 700,000, respectively) induces strong flocculation on fine chlorite, and the CMC with a higher molecular weight gives stronger flocculation. In addition, Figure 8 indicates that the two types of lower molecular weight CMC have a strong dispersing effect at the lowest dosage. This is complementary to the rise in flotation recovery at the same concentrations presented in Figure 7.

**Figure 9:**
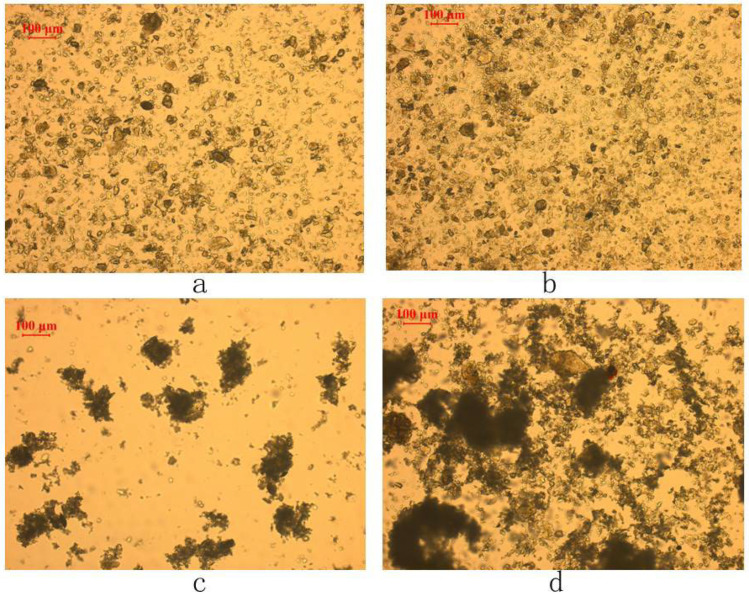
Flocculation/dispersion state of −38 μm chlorite in the presence of different types of CMC with condition of *C* (CMC) = 20 mg/L, pH = 9.0 ± 0.2: (**a**) in the absence of CMC, (**b**) with the addition of CMC with molecular weight of 90,000, (**c**) with the addition of CMC with molecular weight of 250,000, and (**d**) with the addition of CMC with molecular weight of 700,000.

Figure 9, which is composed of four images taken with a polarized optical microscope under different conditions, is evidence of the flocculation/dispersion of the fine chlorite. The images confirm that when conditioned without CMC or in the presence of the CMC with a molecular weight of 90,000, the fine chlorite particles are in a good state of dispersion. However, with the addition of the CMC with a high molecular weight of 250,000 and 700,000, respectively, the sizes of the fine chlorite particles are enlarged, which indicates that flocculation occurs on the fine chlorite particles. In addition, we can see clearly that the CMC with a higher molecular weight results in stronger flocculation, as mentioned above.

These results reveal that the CMC with a low molecular weight almost does not affect the settling behavior of the fine chlorite and is not an effective depressant for its failure in reducing froth entrainment in flotation, despite the fact that it can render the fine chlorite particles hydrophilic. On the other hand, the CMC with a high molecular weight, which generates flocculation on the fine chlorite particles, gives a low flotation recovery of the fine chlorite because of the reduction of froth entrainment. Thus, the conclusion can be drawn that froth entrainment is an important factor for the difference between the low molecular weight CMC and the high molecular weight CMC in the depression of fine chlorite. In addition, the flocculation caused by the high molecular weight CMC is significant in reducing the froth entrainment of fine chlorite particles, which is consistent with the findings suggested by Liu et al. [23].

## 4. Conclusions

This study systematically investigated the influence of CMC with different molecular weights on the flotation of chlorite. Flotation results indicate that the recovery of fine chlorite increases with particle size reduction due to the entrainment through water recovery. However, a high molecular weight CMC is found to be more effective in depressing the flotation of fine chlorite than a low molecular weight one. Sedimentation tests and microscope analysis show that the flocculation/dispersion state plays an important role in the depression effect of CMC on the flotation of fine chlorite. A high molecular weight CMC can flocculate fine chlorite particles to reduce froth entrainment as well as hydrophily, while a low molecular weight CMC fails. It is suggested that the reduction of entrainment because of flocculation caused by CMC is key to realizing the depression of fine chlorite flotation, which also provides a reference for the flotation of other fine minerals.

## Figures and Tables

**Table 1:** Chemical compositions of chlorite.

Composition	MgO	SiO_2_	Al_2_O_3_	CaO	TFe
Percentage/%	25.41	34.37	15.76	0.78	3.72
